# Atypical hemangioma mimicking mixed hepatocellular cholangiocarcinoma: Case report

**DOI:** 10.1097/MD.0000000000009192

**Published:** 2017-12-15

**Authors:** Shengzhang Lin, Lele Zhang, Mengxia Li, Qiyang Cheng, Liang Zhang, Shusen Zheng

**Affiliations:** a State Collaborative Innovation Center for Diagnosis and Treatment of Infectious Diseases, First Affiliated Hospital, College of Medicine, Zhejiang University; b Key Lab of Combined Multi-Organ Transplantation, Ministry of Public Health, Key Lab of Organ Transplantation; c Department of Hepatobiliary and Pancreatic Surgery, First Affiliated Hospital, Zhejiang University School of Medicine, Hangzhou, China.

**Keywords:** atypical hepatic hemangioma, computed tomography, mixed hepatocellular cholangiocarcinoma, magnetic resonance imaging, percutaneous liver biopsy

## Abstract

**Rationale:**

Hemangioma of the liver is a benign hepatic tumor, more common in women than in men, which is typically asymptomatic, solitary, and incidentally discovered. Atypical hemangioma is a variant of hepatic hemangioma with atypical imaging finding features on CT and MRI that can be confused with hepatocellular carcinoma (HCC), intrahepatic cholangiocarcinoma (ICC) and mixed hepatocellular cholangiocarcinoma (HCC-CC).

**Patient concerns:**

We report a case of atypical hepatic hemangioma mimicking HCC-CC: A 59-year-old man was referred to our hospital for a hepatic lesion that was 4.7×3.6 cm in size and located in segments 2 and 3 of the liver. Serum alpha-fetoprotein (AFP) level increased from 17.03 ng/mL to 374.9 ng/mL. The patient was positive for hepatitis B.

**Diagnoses:**

Atypical hepatic hemangioma.

**Interventions:**

US, CT, MRI and Tc-99m RBC liver scans were performed. Dynamic contrast-enhanced MRI showed no uptake in the corresponding area during the arterial phase, peripheral nodular enhancement during the portal phase and delayed phase, and hypo-intensity in the central area. An initial diagnosis of HCC-CC was offered based on the history and imaging findings. Finally, percutaneous liver biopsy (PLB) was offered to the patient. Histopathologic examination of the liver lesions revealed nodular cirrhosis and atypical hyperplasia of liver cells with cavernous hemangioma, where numerous old Schistosoma japonicum eggs were found.

**Outcomes:**

Accurate diagnosis of the patient obviated the need for surgery. The patient's recovery after liver puncture was uneventful, and he was discharged on the seventh post-operative day.

**Lessons:**

In some cases, accurate preoperative imaging of focal hepatic lesions is essential but insufficient for diagnosis. PLB and histopathological examination are important, especially in patients with suspected malignancy.

## 1 Introduction

Hepatic hemangioma is the most common benign neoplasm of the liver. Patients with this condition are typically asymptomatic. The prevalence of hepatic hemangiomas ranges from 1% to 20% in the general population.^[1,2]^ Hepatic hemangioma is primarily diagnosed via imaging studies or autopsies and mainly presents in middle-aged females. Cavernous hemangioma is a common type of hepatic hemangioma, which usually presents as a solitary, well-delineated, subcapsular, disclosed nodule. These distinctive structures show a characteristic hemodynamic pattern on enhanced computed tomography (CT) and magnetic resonance imaging (MRI). However, an atypical hemangioma may lack the imaging features that are characteristic of a typical hemangioma. Hepatic hemangioma can therefore be difficult to distinguish from other lesions such as hepatocellular carcinoma (HCC), intrahepatic cholangiocarcinoma (ICC), and mixed hepatocellular cholangiocarcinoma (HCC-CC). We describe a case of atypical hemangioma mimicking HCC-CC that was misdiagnosed before percutaneous liver biopsy (PLB).

## 2 Materials and methods

The pathology record was retrieved from the Department of Pathology, First Affiliated Hospital, School of Medicine, Zhejiang University. The study was reviewed and approved by the Institutional Review Board of First Affiliated Hospital, School of Medicine, Zhejiang University.

## 3 Case report

A 59-year-old male patient presented with a habit of heavy drinking (700 mL/day) for more than 40 years, history of atrial fibrillation for approximately 6 years, and a 28-year history as a known carrier of Hepatitis B (HBV). He had consulted a doctor for weakness and dizziness 2 months earlier. Entecavir and compound glycyrrhizin capsule therapy were administered immediately. The serum level of aspartate aminotransferase (ALT) was 142 U/L (normal range, 5–40 U/L), and alpha-fetoprotein (AFP) was 17.03 ng/mL (normal, <20 ng/mL). Serum HBV DNA was detected, and the viral load reached 4.7 × 10^5^ copies/mL. Plain CT revealed a space-occupying lesion in segments 2 and 3 of the liver, which was suspected to be a liver neoplasm.

A laboratory workup on admission showed that the serum level for total bilirubin was 27 μmol/L with 18 μmol/L of unconjugated bilirubin; ALT was 46 IU/L; alanine aminotransferase (AST) was 42 ng/mL; laminin was 64.5 ng/mL (normal range, 0.0–50.0 ng/mL); albumin/globulin ratio was 1.1 (normal, 1.5–2.5); and alpha-fucosylated glycosidase was 54 U/L (normal, 10–35). All other liver function tests were within normal limits. Serum AFP increased to 374.9 ng/mL (normal, <20 ng/mL), and serum ferritin was 789.6 ng/mL. Other tumor markers, including carcinoembryonic antigen, carbohydrate antigen 125, carbohydrate antigen 199 (CA199), and prostate-specific antigen, were within normal limits. Repeat testing for HBV DNA was negative; serum hepatitis B virus surface antigen (dilution) was 2444.80 IU/L; and anti-hepatitis B virus core antibody was 12.85S/C.

**Figure 1:**
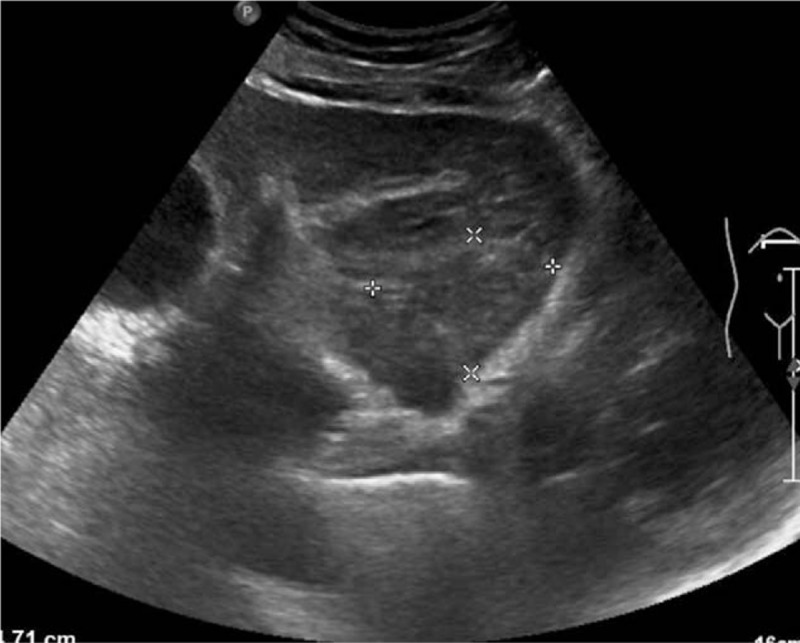
Abdominal ultrasonography. A well-defined, heterogeneously hypo-echoic mass (4.7×3.6 cm in size) was found in the left lateral liver. The boundary and vascular network were not clearly defined.

Abdominal ultrasonography (US) revealed multiple cystic dark regions in the liver. The largest was 9.5 mm in size with thin walls and an enhancement effect. A well-defined, heterogeneously hypo-echoic mass (4.7 × 3.6 cm) was found in the left, lateral liver (Fig. 1). The boundary and vascular network were not clear. There was no obvious abnormality in the spleen, pancreas, or gallbladder.

**Figure 2:**
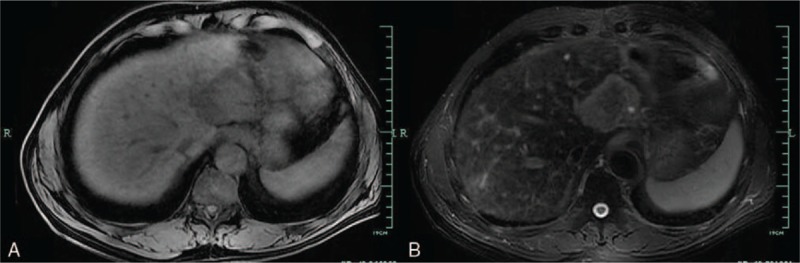
Magnetic resonance imaging shows a lesion with slightly low-signal intensity on T1-weighted imaging (A) and slightly high signal intensity on T2-weighted imaging (B).

**Figure 3:**
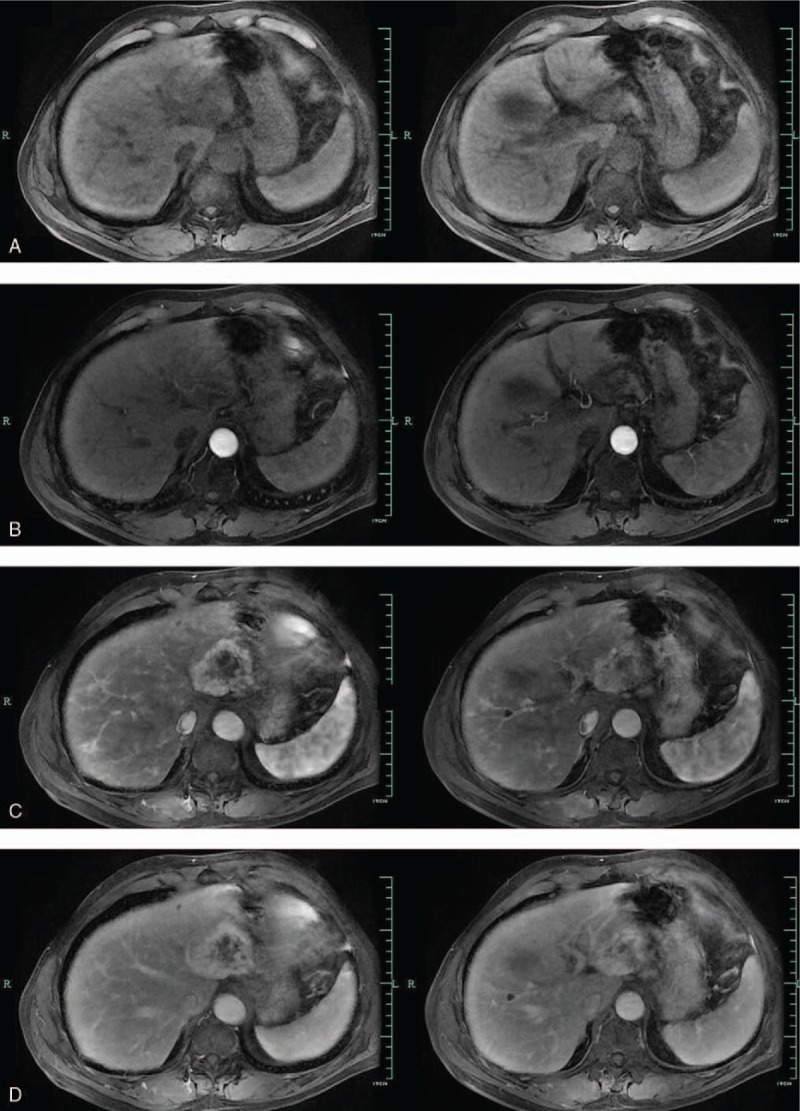
Dynamic contrast-enhanced Magnetic resonance imaging (MRI) scans. (A) Precontrast MRI scan shows a large, heterogeneous mass with low signal intensity in liver. (B) Arterial phase of dynamic, contrast-enhanced MRI scan showed no obvious uptake. (C and D) Portal phase and delayed phase of MRI scans showing gradual progressive centripetal enhancement from peripheral to central areas, except for central areas of low signal intensity.

MRI showed that the well-marginated lesion (5.6 × 5.9 cm, in segments 2 and 3 of the liver) had slightly low signal intensity on T1-weighted images and slightly high signal intensity on T2-weighted images (Fig. 2). Magnetic resonance diffusion-weighted imaging revealed slightly high signal intensity with nonuniform internal signal. Dynamic contrast-enhanced MRI showed no obvious uptake in the corresponding area during the hepatobiliary phase or the arterial phase. The image showed progressive opacification from the periphery to the center, then presented nodular enhancement during the portal and delayed phases; the central area demonstrated hypo-intensity. Based on these features, we considered the lesion to be a liver neoplasm (Fig. 3).

**Figure 4:**
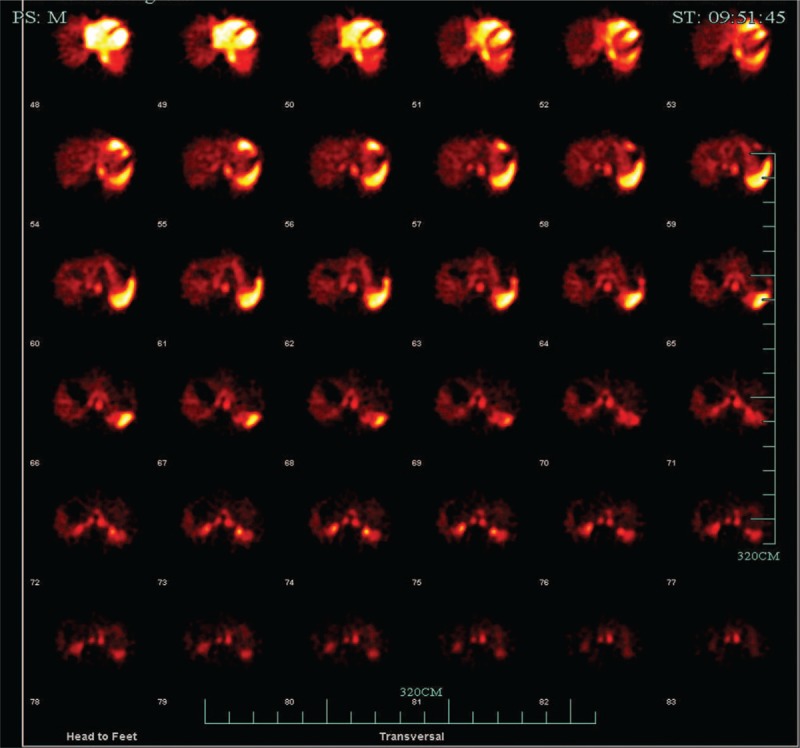
Technetiu-99m–labeled red blood cell scintigraphy.

To rule out the possibility of a hemangioma, Tc-99m red blood cell (RBC) SPECT was performed beginning 60 minutes after the injection of Tc-99m RBC. Liver blood pool tomography revealed a round area of homogeneously increased radioactivity in the left hepatic lobe proximal to the hilus hepatis, except in the central area. The result suggested atypical hemangioma (Fig. 4).

An initial diagnosis of HCC-CC was offered based on the history and imaging findings. The imaging findings were highly suggestive of ICC. However, the patient's clinical symptoms were not obvious. The patient lacked the following symptoms of biliary tract obstruction: jaundice, weight loss, abdominal pain, fever, pruritus. The level of CA199 was within the normal limit.^[3]^ Meanwhile, the fluctuation of viral load and the high level of AFP suggested HCC. Based on the patient's history of HBV, the possibility of acute exacerbations of chronic hepatitis B could not be ruled out.

**Figure 5:**
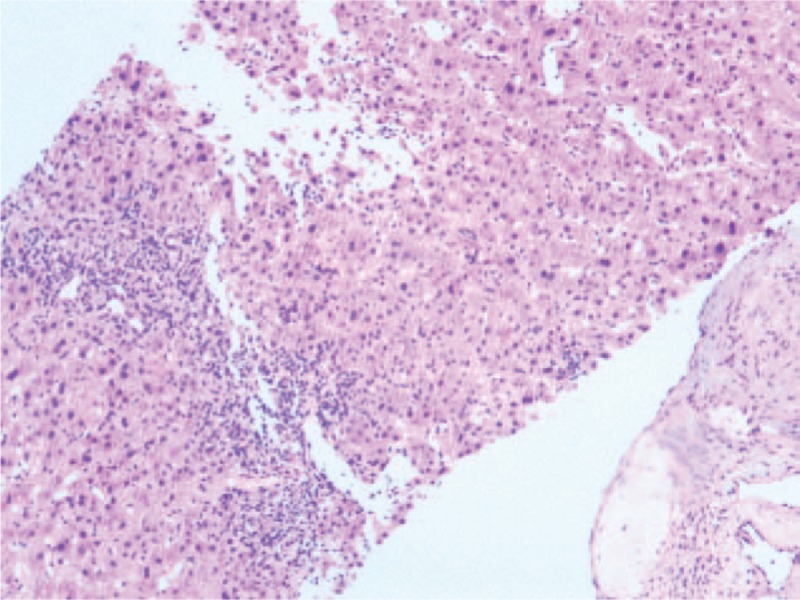
Microscopic examination of liver lesion tissue; (H&E, ×50) Nodular cirrhosis.

**Figure 6:**
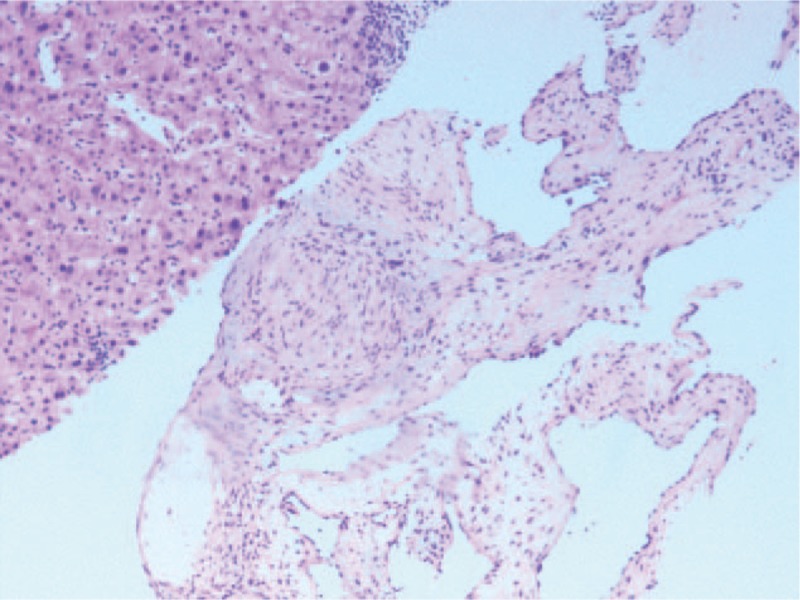
Microscopic examination of liver lesion tissue; (H&E, ×50) Cavernous hemangioma.

**Figure 8:**
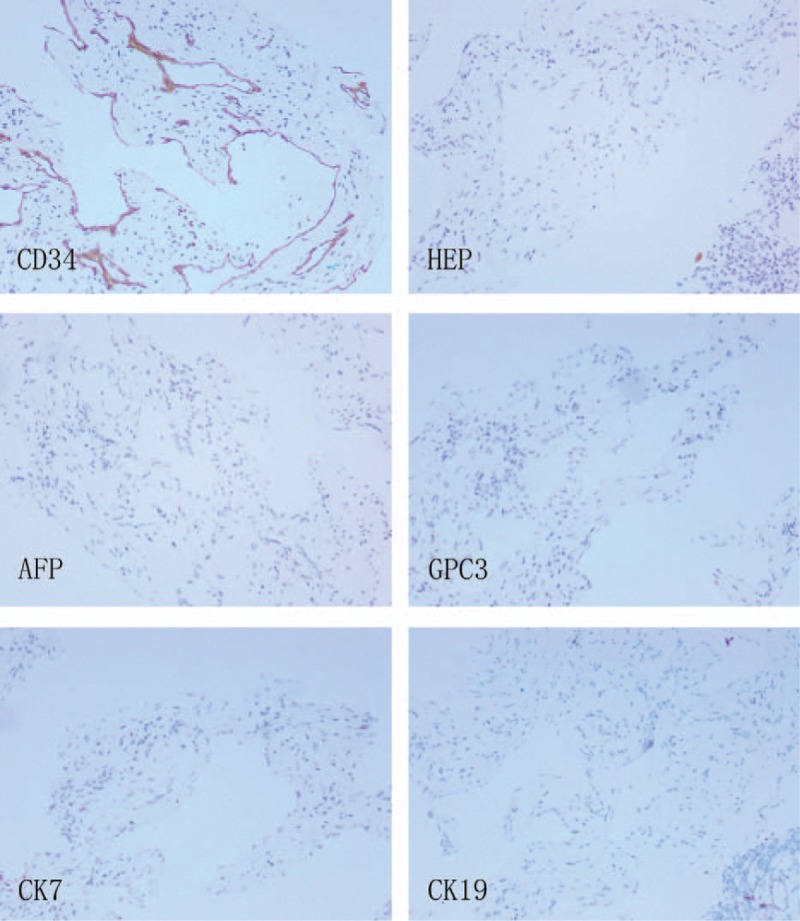
Immunohistochemical staining of the liver hemangioma. Immunohistochemistry revealed positive for cluster of cluster of differentiation 34 and hepatocyte paraffin, but negative for alpha-fetoprotein, glypican-3, cytokeratin 19 and cytokeratin 7 (×100).

To confirm the diagnosis, the patient underwent an ultrasound-guided percutaneous biopsy of the liver lesion. Histopathological examination of the liver lesions revealed nodular cirrhosis (Fig. 5) and atypical hyperplasia of liver cells with cavernous hemangioma (Fig. 6), where numerous old *Schistosoma japonicum* eggs were found (Fig. 7). In addition, in the cavernous hemangioma, the results of immunohistochemistry revealed positivity for a cluster of differentiation 34 and hepatocyte paraffin, negative for AFP, cytokeratin 19, cytokeratin 7, and glypican-3 (Fig. 8).

**Figure:**
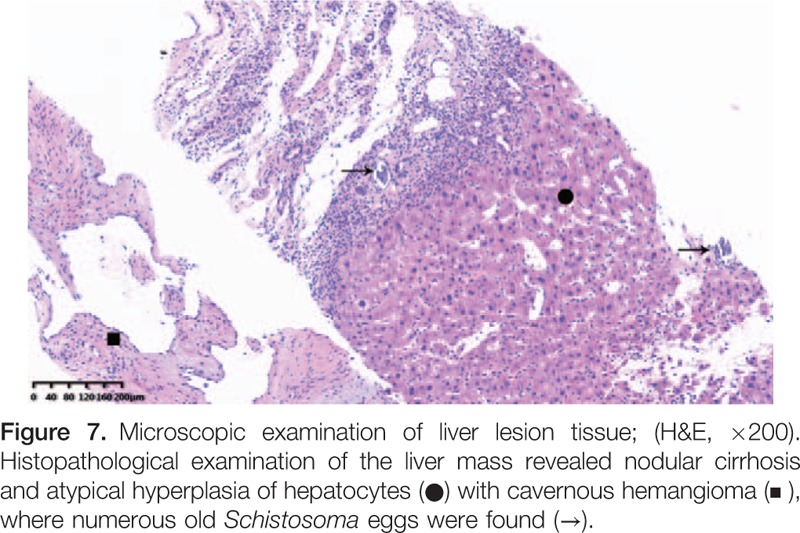


**Figure 9:**
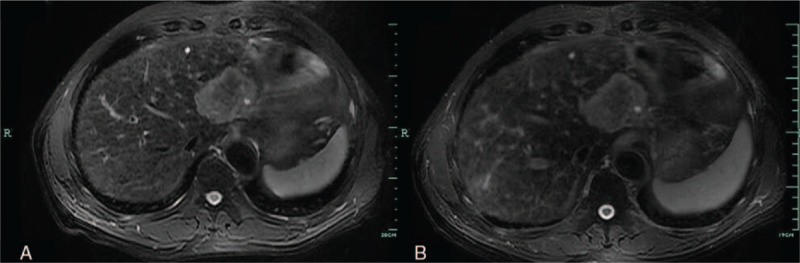
Comparison of magnetic resonance imaging obtained on admission (A) and after 2 months (B).

Based on the results of the histopathological examination, the final diagnosis was nodular cirrhosis with atypical cavernous hemangioma. This diagnosis allowed the patient to avoid unnecessary surgery for hepatic resection. The patient's recovery after liver puncture was uneventful, and he was discharged on the seventh postoperative day. On review after 2 months, he was well, and the MRI reexamination showed that the lesion had not significantly changed (Fig. 9).

## 4 Discussion

Hemangioma of the liver is the most common benign hepatic tumor in the general population. The condition is more common among women. Most patients are asymptomatic with a solitary tumor, which is found incidentally and may readily be diagnosed via characteristic imaging findings: homogenous hyperechogenic mass on US; hypo-attenuating lesions on plain CT; nodular peripheral enhancement during the arterial phase; progressive enhancement from periphery to the center; eventual isodense fill-in on delayed contrast scan during CT or MRI; and “light-bulb”-like high signal intensity on heavily T2-weighted MRI images.^[4]^ Overall, CT and MRI are necessary for the diagnosis of hepatic hemangioma.^[5]^

Sometimes, benign lesions can deceive doctors by appearing as malignant tumors on imaging. For example, hemangioma can exhibit various forms of pathology, depending on the co-occurrence of hepatitis virus, bilharziasis, thrombosis, and others. The associated imaging findings are easily confused with primary or metastatic malignancy.^[6]^ Even MRI has a sensitivity and specificity of >90%; only 50% of all hemangiomas yield typical radiological findings.^[7]^ Thus, for some atypical hemangiomas, imaging findings are not always correct. Furthermore, when the patient concomitantly had high AFP, which is a risk factor for tumor malignancy, we should attribute the symptoms to a single disease: HCC.

Similar to the case we reported, 1 quinquagenarian patient had a long history of HBV without therapy. His laboratory data showed serum levels of AFP that had increased from 17.03 to 374.9 ng/mL in 2 months. The history and laboratory findings suggested a diagnosis of HCC, but we could not rule out the possibility of acute exacerbations of chronic hepatitis B.

Additionally, dynamic contrast-enhanced MRI showed progressive opacification from the periphery to the center during the portal phase and delayed phase, with peripheral nodular enhancement; this is a typical imaging feature of ICC.^[8]^ Early painless jaundice is the main sign in most ICC patients; the patient's serum bilirubin levels were only slightly elevated, and levels of alkaline phosphatase were within normal limits. Although CA199 and CK-19 are not specific markers for disease, they can assist in the diagnosis of ICC. In the patient presented here, tests for CA199 and CK-19 yielded negative results.

To rule out the possibility of a benign lesion, a Tc-99m RBC liver scan was performed, but the result did not conform to typical hemangioma. Generally, a Tc-99m RBC liver scan has proven to be a useful means of differentiating benign lesions from malignant lesions. The characteristic feature of hepatic hemangioma in images is relatively reduced flow to the lesion compared with the liver during dynamic study, with complete filling in the delayed image.^[9]^

US, CT, MRI, and Tc-99m RBC liver scans are helpful tools in the clinical diagnosis of hepatic lesions. According to the above features, a clinical diagnosis of HCC-CC was made. The lesion was diagnosed as a rare primary hepatic neoplasm with hepatocellular and biliary features, requiring surgical extirpation.^[10]^

To verify this diagnosis, we performed PLB and histopathological examination of the liver lesion. As we know, the gold standard of diagnosing lesions is pathological diagnosis, which requires liver resection or PLB. PLB is an important tool for the diagnosis and treatment of liver lesions. This approach provides tissue for histopathological observation. However, as an invasive procedure, PLB carries a risk for certain complications, such as abdominal dissemination by needle-tract implantation.^[11]^ Nonetheless, PLB is simple, relatively safe, and often accurate in diagnosis. The technique can be commonly performed in modern hepatology.

Interestingly, histopathological examination revealed nodular cirrhosis and atypical cavernous hemangioma. Accurate diagnosis of the patient obviated the need for surgery, which may cause serious complications and sequelae for a 56-year-old man in a weakened condition.

Given the findings described above, a controversial problem emerges: how do we account for the elevated AFP? AFP is a kind of fetal-specific glycoprotein produced by the fetal liver, which has a normal range in adults of less than 20 ng/mL. Serum AFP is elevated in cases such as HCC, seminoma, and pancreatic cancer. According to the conclusions of the Barcelona-2000 EASL Conference (EASL: European Association for the Study of the Liver), the diagnostic criteria of HCC include AFP levels >400 ng/mL and focal lesion >2 cm with arterial hypervascularization.^[12]^ Meanwhile, false-positive results should also be considered. For instance, increased serum AFP may be detected in patients with drug abuse, alcoholic cirrhosis and other states of chronic liver damage. However, AFP levels under these conditions are usually <100 ng/mL,^[13]^ which can be separated from HCC.

In our case, repeated inspection results of AFP were significantly abnormal: false-positive results could be excluded. The immunohistochemistry analysis of PLB demonstrated that AFP was negative in the lesion, which contradicted the continuous high serum AFP level. Therefore, combining the long history of HBV with the clinical manifestations of weakness and dizziness, we presumed that the nonmalignant serum AFP elevation was caused by the acute exacerbation of chronic hepatitis B. Recently, this conjecture was supported by strong evidence: the latest level of AFP was down to 22.0 ng/mL, close to normal.

MRI reexamination (Fig. 9) after 2 months showed no significant changes in the lesion, which further strengthened the diagnosis. Although the cavernous hemangioma was a benign lesion, and the PLB revealed no sign of hepatic malignancy, such lesions may change over time. Unlike other ordinary patients with typical hemangiomas, the patient suffered from chronic hepatitis B and had a long-standing habit of heavy drinking, 2 independent risk factors for liver cirrhosis and HCC. To avoid lesion deterioration, the patient must therefore be intensively followed up with imaging and laboratory investigations. This case confirms the importance and necessity of percutaneous biopsy and histopathological examination. Additionally, mild or moderately high AFP levels could be seen in the benign hepatic lesions, which may easily and wrongly suggest malignant liver tumors, so it is necessary to detect dynamic changes in AFP that is elevated.

## 5 Conclusion

Here, we report a case of atypical hepatic cavernous hemangioma in an HBV carrier with high AFP. The lesion was benign but required differentiation from hepatic malignancies. However, in some cases, accurate preoperative imaging of focal hepatic lesions is essential but insufficient for diagnosis. Relying exclusively on preoperative imaging could lead to misdiagnosis or unnecessary hepatic resection. PLB and histopathological examination are important, especially in patients with suspected malignancy.
